# Purification and characterization of a novel neutral and heat-tolerant phytase from a newly isolated strain *Bacillus nealsonii* ZJ0702

**DOI:** 10.1186/1472-6750-13-78

**Published:** 2013-09-28

**Authors:** Ping Yu, Yirun Chen

**Affiliations:** 1 College of Food Science and Biotechnology, Zhejiang Gongshang University, 149 Jiaogong Road, Hangzhou 310035, Zhejiang Province, PR China

**Keywords:** Phytase, Purification and characterization, Heat-tolerant, Homology analysis, *Bacillus nealsonii*

## Abstract

**Background:**

Phytic acid and phytates can interact with biomolecules, such as proteins and carbohydrates, and are anti-nutritional factors found in food and feed. Therefore, it is necessary to remove these compounds in food and feed processing. Phytase can hydrolyze phytic acid and phytates to release a series of lower phosphate esters of myoinositol and orthophosphate. Thus, the purification and characterization of novel phytases that can be used in food and feed processing is of particular interest to the food and feed industries.

**Results:**

A novel neutral and heat-tolerant phytase from a newly isolated strain *Bacillus nealsonii* ZJ0702 was purified to homogeneity with a yield of 5.7% and a purification fold of 44. The molecular weight of the purified phytase obtained by SDS-PAGE was 43 kDa. The homology analysis based on N-terminal amino acid and DNA sequencing indicated that the purified phytase was different from other known phytases. The optimal thermal and pH activity of the phytase was observed at 55°C and 7.5, respectively. Seventy-three percent of the original activity of the phytase was maintained following incubation at 90°C for 10 min. The phytase was stable within a pH range of 6.0 − 8.0 and showed high substrate specificity for sodium phytate. Cu^2+^, Co^2+^, Zn^2+^, Mn^2+^, Ba^2+^ and Ni^2+^ ions were found to inhibit the activity of the phytase.

**Conclusions:**

A novel phytase purified from *B. nealsonii* ZJ0702 was identified. The phytase was found to be thermally stable over a wide temperature range at neutral pH. These properties suggest that this phytase is a suitable alternative to fungal phytases for the hydrolysis of phytic acid and phytates in food and feed processing industries.

## Background

Phytic acid and phytates exist widely in edible legumes, cereals, oil seeds, pollens and nuts, and account for about 1 − 5% of the dry weight of plant seeds [1,2]. The presence of phytic acid and phytates in plant food and feed has been well documented [3-6]. They are a primary source of inositol and an important storage form of phosphorus in plant seeds that are often used as animal feed ingredients [7-9]. Because of their ability to interact with biomolecules, such as proteins and carbohydrates, they can act as anti-nutritional factors in several ways: (i) chelating cations, such as Ca^2+^, Mg^2+^, Fe^2+^ and Zn^2+^, to form insoluble metal phytate complexes under gastrointestinal pH conditions; (ii) reducing the digestibility of protein, starch and lipids; and (iii) inhibiting the activity of enzymes, including amylase, trypsin, acidic phosphatase and tyrosinase [2,10-15]. Thus, it is necessary to remove phytic acid and phytates in food and feed processing to avoid the above-mentioned problems.

Phytase (myoinositol hexakisphosphate phosphohydrolases EC3.1.3.8) cleaves phosphor- monoester bonds in phytic acid and phytates. This results in the sequential release of a series of lower phosphate esters of myoinositol and orthophosphate. Therefore, phytase has gained rapid acceptance as an animal feed or a food additive worldwide [16]. In addition, phytase has important applications in ameliorating human nutrition [17-19], as well as in some other areas, including aquaculture [20]. Thus, it is highly desirable to reduce the content of phytic acid and phytates in food and feed processing by the hydrolysis of phytase.

A wide variety of phytases have been isolated from different organisms [21-31]. However, the focus has been on fungus-derived phytases that are active at low pH values and show low thermal stability [31-34]. Moreover, although the addition of phytase is widely used to improve the release of plant phosphorus in poultry and swine, the use of phytase in feed for aquatic species has not been developed [35]. Some aquaculture species are agastric and their digestive system pH is neutral. Thus, fungus-derived acidic phytases are of limited use to these species because these phytases show low activity at neutral pH. Bacterial phytases are an important alternative to fungal ones because of their high thermal stability, phytate substrate specificity, wide pH profile and proteolysis resistance [33,36,37]. Therefore, the purification and characterization of novel phytases with high thermal stability and activity at neutral pH from bacteria is of particular interest for future industrial applications in food and feed processing.

In the present study, a novel neutral and heat-tolerant phytase from a newly isolated strain *Bacillus nealsonii* ZJ0702 from the soil was sequentially purified to homogeneity by ammonium sulfate precipitation, DEAE-sepharose Fast Flow column chromatography and Sephadex G-100 size-exclusion chromatography. The enzymatic properties of the purified phytase were investigated in detail.

## Results

### Purification of the phytase from *B. nealsonii* ZJ0702

**Figure 1:**
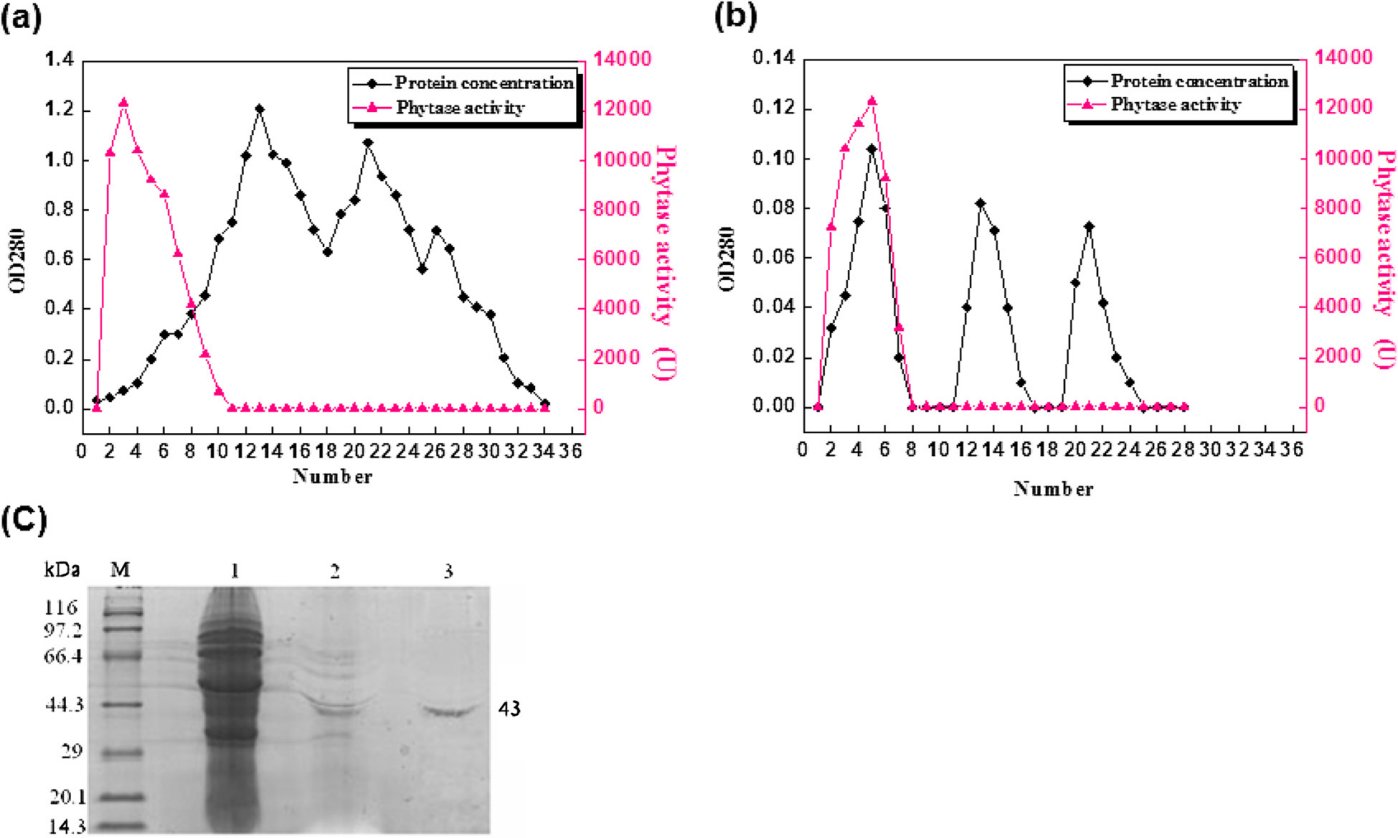
**Elution curves of the phytase from *B. nealsonii *ZJ0702 and the SDS-PAGE analysis. (a)** The elution curve of the DEAE-sepharose anion-exchange column chromatography purification step of the sample. **(b)** The elution curve of the Sephadex G-100 size-exclusion chromatography purification step of the sample. **(c)** The SDS-PAGE analysis of the phytase. M: protein molecular weight markers; lane 1: the sample from the crude extract; lane 2: the sample after DEAE-sepharose anion-exchange column chromatography; lane 3: the sample after Sephadex G-100 size-exclusion chromatography.

The supernatant obtained by centrifugation of the culture broth at 12,000rpm for 20 min was used as the enzyme source. The purification of the phytase was sequentially performed by (NH_4_)_2_SO_4_ precipitation, DEAE-sepharose anion-exchange column chromatography and Sephadex G-100 size-exclusion column chromatography. The purification results are presented in Figure 1 and Table 1. After (NH_4_)_2_SO_4_ precipitation, 76% of the total protein was removed. The residual protein was subject to DEAE-sepharose anion-exchange column chromatography (Figure 1a), and the protein containing the activity of phytase was collected and pooled, giving 6% of the total protein. The protein was subject to further purification by Sephadex G-100 size-exclusion chromatography. Here, fractions 2–8 showed the highest phytase activity (Figure 1b). These fractions were collected and pooled, giving 1% of the total protein. The purification folds of phytase from the above three purification steps were 2, 10 and 44, respectively. Corresponding recovery rates of the total activity of phytase were 49, 6 and 5.7%, respectively (Table 1). The SDS-PAGE analysis of the protein samples from the above three purification steps is shown in Figure 1c. Only a single protein band with an estimated molecular weight of 43 kDa was present following the Sephadex G-100 size-exclusion chromatography step. This result indicates that the obtained phytase is electrophoretically pure and can be used for the analysis of enzymatic properties.

**Table 1:** **Purification results of the phytase from a newly isolated strain *B. nealsonii *ZJ0702**

**Purification steps**	**Total protein**	**Total activity**	**Specific activity**	**Purification fold**	**Yield**
	**(mg)**	**(U)**	**(U/mg)**		**(%)**
Culture broth	101.7	8760001	8611	1	100
(NH_4_)_2_SO_4_ precipitation	24.7	4300001	17430	2	49
DEAE-sepharose Fast Flow	6	526240	87120	10	6
Sephadex G-100	1	501764	380124	44	5.7

### Homology analysis of the phytase

**Figure 2:**
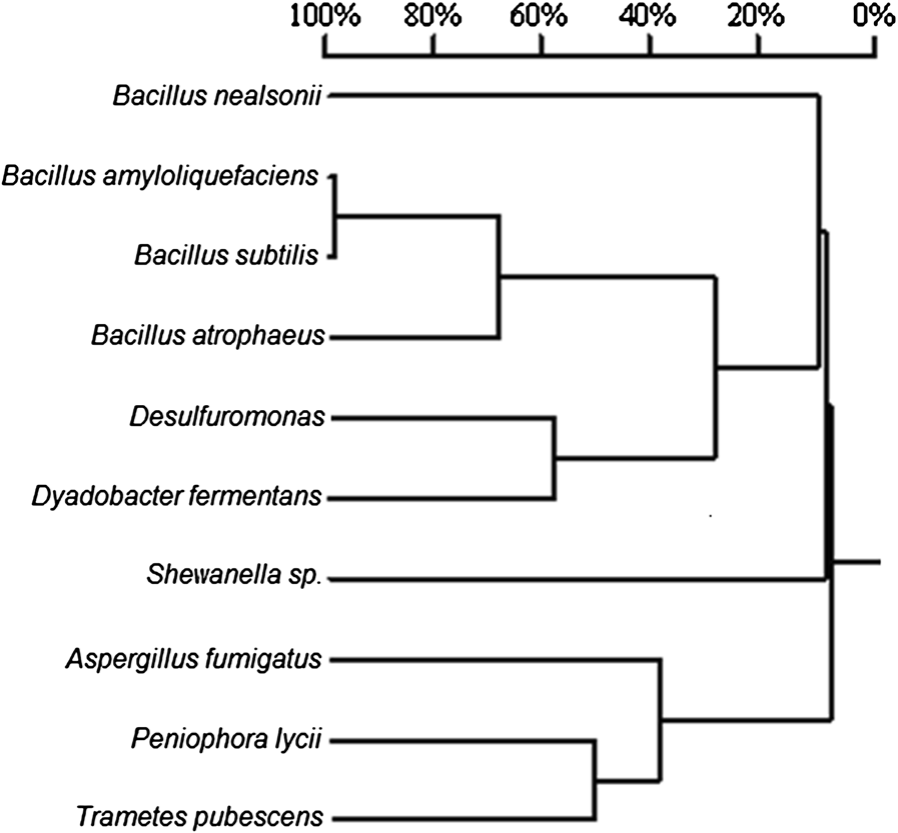
Polygenetic tree of phytases based on DNA sequences.

The homology tree of phytase based on the DNA sequence is presented in Figure 2. A very low sequence homology was found between the DNA sequence of the phytase from *B. nealsonii* ZJ0702 and phytases from selected microorganisms. The determined N-terminal amino acid sequence of the purified phytase from *B. nealsonii* ZJ0702 is: MGAIDTCPNKYSTIRRVLIMN KKTQMIHGGH. A similarity comparison was also carried out between this protein sequence and the corresponding regions of other known phytases; however, no similarity was found. These results strongly suggest that the phytase from *B. nealsonii* ZJ0702 is different from other known phytases (Figure 2).

### Enzymatic properties of the purified phytase

**Figure 3:**
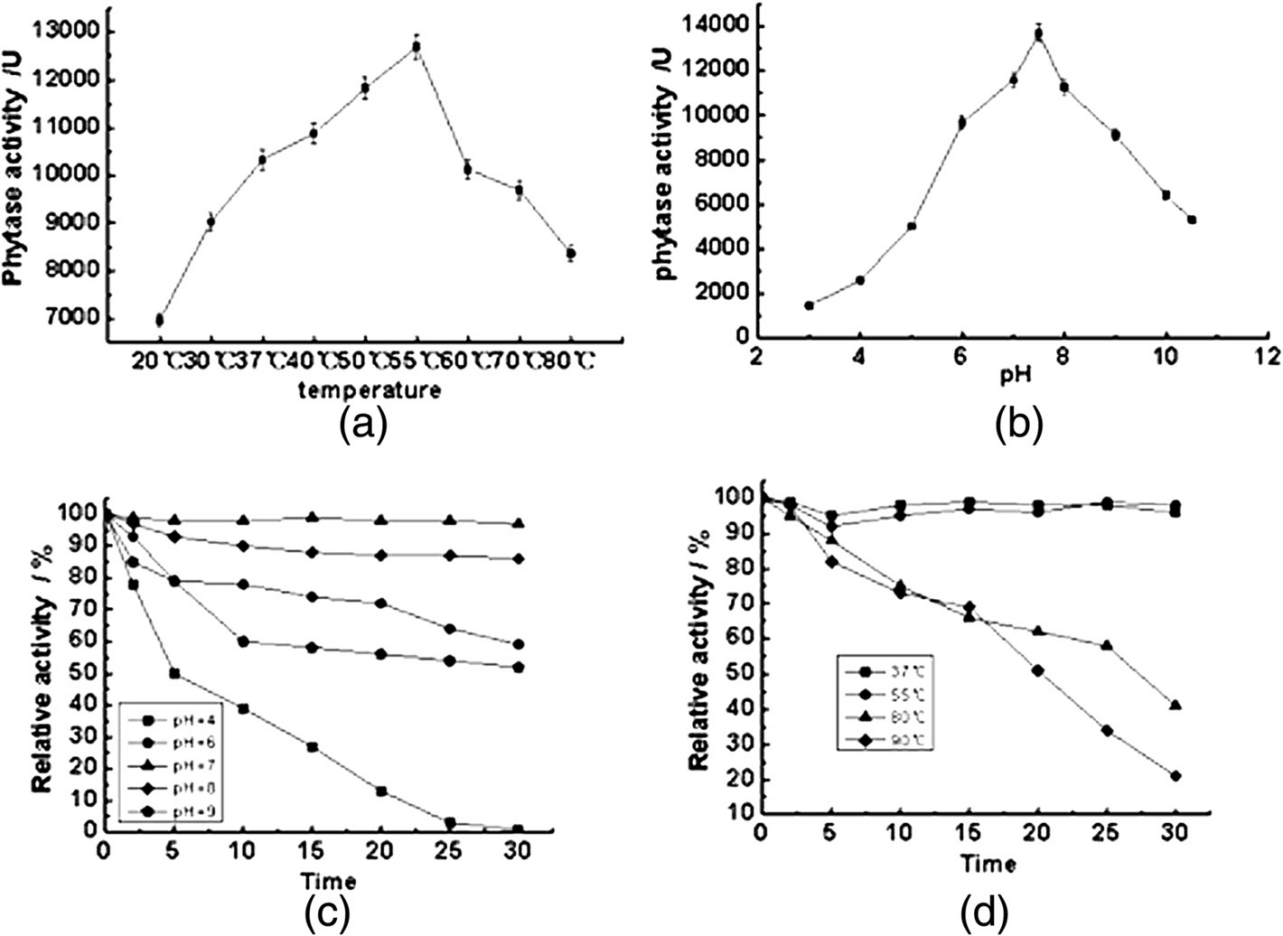
**Enzymatic properties of the purified phytase. (a)** Effect of temperature on the activity of the phytase. **(b)** Effect of pH on the activity of the phytase. **(c)** The thermal stability of the phytase. **(d)** pH stability of the phytase. The optimal temperatures for the activity of phytase were investigated by incubating 0.5 ml reaction mixture for 10 min. The reaction mixtures contained 0.2 ml of 20 mM phosphate buffer (pH 7.0), 0.2 ml sodium phytate as the substrate and 0.1 ml of the phytase, and the temperature range examined was 20–80°C. The effect of pH on the activity of the phytase was studied using 0.5 ml reaction mixtures containing 0.2 ml of the buffer and 0.2 ml sodium phytate as the substrate at 55°C for 10 min. Buffers used were: 0.1 M glycin − HCl buffer, pH 3.0; 0.1 M acetic acid buffer, pH 4.0 − 5.0; 0.1 M Tris–HCl buffer, pH 6.0 − 9.0; and 0.1 M glycine − NaOH buffer, pH 10.0 − 11.0. To study the stability of the phytase, aliquots of the enzyme solutions were subject to different temperatures and pHs for 30 min. Temperatures used were 37, 55, 80 and 90°C. pHs used were 4, 6, 7, 8 and 9. The residual activity of the phytase was detected once every 5 min, and the relative activity of the phytase was calculated. The control sample is phytase at 4°C and pH 7.0, and its activity is defined as 100%.

Enzymatic properties of the purified phytase are shown in Figure 3. The activity of the phytase increased when the temperature was increased from 20 to 50°C, and reached a maximal value at 55°C. Thereafter, it decreased rapidly as the temperature increased beyond 55°C. This shows that the optimal temperature of the purified phytase is 55°C (Figure 3a). The activity of the phytase varied as a function of the pH. The highest activity of the phytase was observed at pH7.5 (Figure 3b). For the phytase from *B. nealsonii* ZJ0702, strong thermal stability was observed at 37 and 55°C. The activity of the phytase showed negligible change when incubated at either of these temperatures for 30 min. The residual activities of the phytase were 75, 62 and 41% when the protein was incubated at 80°C for 10, 20 and 30 min. The residual activities of the phytase at 90°C were 73, 51 and 21% when the protein was incubated for 10, 20 and 30 min, respectively (Figure 3c). A high pH stability of the phytase was observed when it was incubated at pH7.0 and 8.0. At pH4.0, the activity of the phytase decreased dramatically as the incubation time increased, and only 1% of the original activity of the phytase remained after incubation at pH4.0 for 30 min (Figure 3d).

The effect of different metal ions on the activity of the purified phytase (Table 2) indicated that Ca^2+^ at 1 mM and 5 mM did not alter its activity. In addition, the activity of the purified phytase was not altered when 1 mMMg^2+^ was added. However, the activity decreased by 38% when the concentration of Mg^2+^ increased to 5 mM. Cu^2+^, Co^2+^, Zn^2+^, Ba^2+^, Mn^2+^ and Ni^2+^ at 1 mM and 5 mM were found to inhibit the activity of phytase significantly. The relative hydrolytic rate of different phosphorylated compounds showed that the purified phytase exhibited very narrow substrate specificity (Table 3). The phytase showed high activity only towards the substrate sodium phytate. No phytase activity was detected when other phosphorylated substrates were used.

**Table 2:** Effect of metal ions on the activity of the purified phytase

**Metal ions**^**a**^	**Relative activity (%)**^**b**^	
	**1 mM**	**5 mM**
Ba^2+^	48	22
Ca^2+^	104	99
Cu^2+^	63	35
Co^2+^	43	21
Mg^2+^	97	62
Mn^2+^	73	51
Ni^2+^	40	20
Zn^2+^	52	28

^a^The counter ion of these metal ions is Cl^−^.

^b^Control samples are phytase solutions without the addition of the metal ions.

**Table 3:** Substrate specificity of the purified phytase

**Substrates**	**Relative activity (%)**
Sodium phytate	100
pNPP	0
Glucose-1-phosphate	0
ATP	0
Fructose-1,6-diphosphate	0
β-Glycerophosphate	0

## Discussion

In this study, a neutral and heat-tolerate phytase from *B. nealsonii* ZJ0702 was purified to homogeneity, and the homology analysis based on N-terminal amino acid sequencing and DNA sequencing revealed that the phytase is novel. This enzyme display the optimal activity at 55°C, which is similar to that of other known phytases from particular microorganisms, such as *Mucor hiemalis* (55°C), *Aspergillus oryzae* (60°C), *Bacillus subtilis* (55–60°C), *Escherichia coli* (55–60°C), *Penicillium simplicissimum* (55°C), *Aspergillus niger* (55–58°C) and *Klebsiella terrigena* (58°C) [38-40]. Compared with acidic phytases from *Saccharomyces cerevisiae* (pH3.6), *Cladosporium* sp. FP-1 (pH4.0), *Pichia anomala* (pH4.0), *Candida krusei* (pH4.6), *Lactobacillus sanfranciscensis* (pH4.0), *Pantoea agglomerans* (pH4.5), *Klebsiella terrigena* (pH5.0) and *Penicillium simplicissimum* (pH4.0) [40-42], the phytase from *B. nealsonii* ZJ0702 showed the optimal activity at pH7.5, indicating that it is neutral and should be suitable for the application in some aquaculture species whose digestive system pH is neutral. The thermal stability of the phytase suggests that it has stronger thermal stability than the phytase from *Yersinia kristensenii*[21]. The strong inhibition of the activity of the phytase by Ba^2+^, Cu^2+^ and Co^2+^ at 5 mM shows that the active site of phytase may include –SH moieties. The high substrate specificity of the phytase for sodium phytate indicates that the phytase from *B. nealsonii* ZJ0702 is highly specific for inositol polyphosphate.

## Conclusions

The results from this study demonstrate that the phytase from *B. nealsonii* ZJ0702 shows optimal activity at neutral pH, strong thermal stability and high substrate specificity for sodium phytate. These unique properties make this phytase an attractive alternative to replace fungus-derived phytases for the hydrolysis of phytic acid and phytates. Extensive studies on the application of the purified phytase in food and feed processing are ongoing.

## Methods

### Strain and reagents

The strain *B. nealsonii* ZJ0702 was isolated from the soil at the Xihu district of Zhejiang Province, China, and was kept in our laboratory. This strain is deposited in the China General Microbiological Culture Collection (CGMCC, No.5396), and produces the extracellular phytase [43]. Phytic acid, sodium phytase, ATP, glucose-1-phosphate, fructose-1, 6-diphosphate, β-glycerophosphate and *p*-nitrophenylphosphate (pNPP), DEAE-sepharose and Sephadex G-100 were purchased from the Sigma Co., Ltd., LA, USA. PCR reagents and the PCR product purification kit were purchased from TaKaRa Biotech Co., Ltd., Japan. The UNIQ-10 DNA extraction kit was purchased from Sangon Co., Ltd., Shanghai, China. The strain culture medium contained 35 g/l wheat bran, 20 g/l tryptone, 5 g/l NH_4_NO_3_, 2 g/l CaCl_2_, 0.5 g/l KCl, 0.5 g/l MgSO_4_ · 7H_2_O and 1 g/l KH_2_PO_4_, pH7.0. The other reagents used in the experiments were of analytical grade and used according to the specifications provided by the manufacturer.

### Strain culture

A single colony of the strain *B. nealsonii* ZJ0702 was transferred from a slant culture to an Erlenmeyer flask (1000 ml) containing 500 ml of the culture medium, followed by incubation at 34°C with vigorous agitation in a shaking incubator at 165 rpm for 72 h for producing the extracellular phytase.

### Enzyme purification

All purification steps were carried out at 4°C unless otherwise stated. The 72 h culture broth was centrifuged at 12,000 rpm for 20 min to remove the cells. The supernatant was collected and ammonium sulfate was first added until 30% saturation. The resultant precipitation was removed by centrifugation at 10,000 rpm for 30 min at 4°C. Proteins were then fractioned from the supernatant by adding ammonium sulfate until different saturation levels were reached (40, 50, 60, 70 and 80%). The obtained precipitates were pelleted by centrifugation at 10,000 rpm for 30 min at 4°C, combined and resuspended in a 0.1 M phosphate buffer (pH7.0). The combined precipitate was desalted using a dialysis bag (diameter: 0.45 μm) and then loaded onto a DEAE-sepharose Fast Flow ion-exchange column. Proteins were eluted at 0.8 ml/min using a phosphate buffer (pH7.0) containing a linear NaCl-gradient with the concentration ranging between 0 and 2 M. The fractions with phytase activity were combined and concentrated by PEG 20,000. The concentrate was loaded onto a Sephadex G-100 chromatography column (2.5 × 35 cm) pre-equilibrated with a phosphate buffer (pH7.0). Proteins were eluted at 0.5 ml/min using the same buffer. The fractions with phytase activity were collected and combined. The protein purity was determined by SDS-PAGE analysis. SDS-PAGE was carried out as described by Laemmli [44].

### Determination of the activity of phytase and the concentration of the total protein

The phytase activity of samples collected from each purification step was analyzed. The phytase activity was determined as described by Engelen et al. [45] with minor modifications. Samples were diluted accordingly before the analysis. 0.5 ml diluted samples and 25 mM sodium phytate in 0.2 M phosphate buffer (pH7.0) were incubated separately at 55°C for 10 min. Then, 0.5 ml of the substrate was added to the sample and the mixture was incubated for another 10 min. Thereafter, 2 ml of 10 mM NH_4_Mo_7_O_24_ · 4H_2_O:5N H_2_SO_4_:acetone (1:1:2) was added. The reaction was allowed to proceed for 30s. The reaction was halted by adding 0.1 ml of 1 M citric acid. The color of the reaction of the Pi (inorganic phosphate)-Mo complex was read at A_380_. A reference standard (KH_2_PO_4_,0.1-0.4 μM) was simultaneously assayed with the samples. A unit (U) of phytase activity was defined as the amount of enzyme required to release 1 nM of Pi per minute at 55°C. The protein concentration was measured according to the Bradford method using bovine albumin as the standard [46].

### PCR amplification of the phytase gene and determination of the N-terminal amino acid sequence

The genomic DNA from *B. nealsonii* ZJ0702 was extracted using the UNIQ-10 DNA extraction kit according to the specifications provided by the manufacturer. The primer set, P_1_: 5′-ATGGGAGCGATCGATACATGTCCAAAC-3′ and P_2_: 5′-TTAGATCGACCCCTGTATGACCACT-3′, was designed for the amplification of the phytase gene by PCR with the genomic DNA as the template. PCR conditions consisted of an initial denaturation at 95°C for 5 min, 35 cycles of the amplification consisted of denaturation at 95°C for 1 min, annealing at 55°C for 1 min and extension at 72°C for 2 min. Then a further extension at 72°C was performed for 10 min. PCR products were purified by the PCR purification kit and sequenced by Sangon Co., Ltd., Shanghai, China. The homology analysis of phytases based on the DNA sequences was carried out with the DNAMAN 7.0 software. For the analysis of the N-terminal amino acid sequence of the purified phytase, the proteins on the SDS-PAGE gel were transferred to a PVDF membrane at 200V for 1 h. After the proteins were stained, the membrane corresponding to the protein band of the purified phytase was cut out and digested with sequencing grade trypsin, as described by Fernandez et al. [47], except that the detergent Triton X-100 was replaced by octyl-β-D- glucopyranoside (Boehringer Mannheim). Phytase was analyzed with a HP G1005A protein automated sequencing system (Hewlett- Packard Co., Ltd.).

### Enzymatic properties of the purified phytase

The temperature stability of the phytase was determined by subjecting aliquots of phytase solutions to different temperatures for 30 min. Temperatures used were 37, 55, 80 and 90°C. The residual activity of the phytase was detected once every 5 min. The effect of temperature on the activity of the phytase was determined at different temperatures ranging from 20 to 80°C. Similarly, the optimal pH for the activity of the phytase was determined by mixing equal volumes of buffers at different pH values ranging from 3.0 to 11.0 at 55°C, while the pH stability was examined by subjecting aliquots of phytase solutions to different pH values for 30 min. The residual activity of the phytase was determined once for 5 min. Buffers used were: 0.1 M glycine-HCl buffer (pH3.0); 0.1 M acetic acid buffer (pH4.0 − 5.0); 0.1 M Tris–HCl buffer (pH6.0 − 9.0); and 0.1 M glycine-NaOH buffer (pH10.0 − 11.0). The phytase sample at 4°C and pH7.0 was used as the control and its activity was defined as 100%. The effect of metal ions on the activity of the phytase was studied by incubating metal ions with a purified enzyme solution (0.5 ml) for 10 min at 55°C. The following metal ions at 1 and 5 mM were used: Ba^2+^, Ca^2+^, Cu^2+^, Co^2+^, Mg^2+^, Mn^2+^, Ni^2+^ and Zn^2+^. The substrate specificity of the purified enzyme was evaluated by following the standard assay procedure, except that the substrate was replaced with different phosphorylated compounds: pNPP, glucose-1-phosphate, ATP, fructose-1,6-diphosphate and β-glycerophosphate.

### Nucleotide sequence accession number

The DNA sequence of the phytase from *B. nealsonii* ZJ0702 is in the GenBank database under accession number HQ843995.

## Competing interests

Both authors declare that they have no competing interests.

## Authors’ contributions

PY designed and guided the experiments and wrote the manuscript. YC carried out the experiments. All authors read and approved the final manuscript.

## References

[B1] Lei XG Porres JM Phytase enzymology, applications, and biotechnology Biotech Lett 2003 25 21 1787 1794 10.1023/A:1026224101580 14677699

[B2] Wodzinski RJ Ullah AHJ Saul LN, Allen IL Phytase Advances in Applied Microbiology 1996 London Academic Press, Inc 263 302 10.1016/s0065-2164(08)70375-7 8865587

[B3] Columbus D Niven SJ Zhu CL de Lange CFM Phosphorus utilization in starter pigs fed high-moisture corn-based liquid diets steeped with phytase J Anim Sci 2010 88 12 3964 3976 10.2527/jas.2010-3011 20729280

[B4] Cowieson AJ Ravindran V Selle PH Influence of dietary phytic acid and source of microbial phytase on ileal endogenous amino acid flows in broiler chickens Poultry Sci 2008 87 11 2287 2299 10.3382/ps.2008-00096 18931180

[B5] Dai F Qiu L Ye L Wu D Zhou M Zhang G Identification of a phytase gene in barley (*Hordeum vulgare* L.) PloS one 2011 6 4 e18829 10.1371/journal.pone.0018829 21533044 PMC3080886

[B6] Farhat A Chouayekh H Ben Farhat M Bouchaala K Bejar S Gene cloning and characterization of a thermostable phytase from *Bacillus subtilis* US417 and assessment of its potential as a feed additive in comparison with a commercial enzyme Mol Biotech 2008 40 2 127 135 10.1007/s12033-008-9068-1 18543132

[B7] Maga JA Phytate: its chemistry, occurrence, food interactions, nutritional significance, and methods of analysis J Agric Food Chem 1982 30 1 1 9 10.1021/jf00109a001

[B8] Harland BF Morris ER Phytate: A good or a bad food component? Nutr Res 1995 15 5 733 754 10.1016/0271-5317(95)00040-P

[B9] Graf E Phytic acid: chemistry and applications 1986 USA California: Pilatus Press

[B10] Lee DY Schroeder J Gordon DT Enhancement of Cu bioavailability in the rat by phytic acid J Nutr 1988 118 6 712 717 3373335 10.1093/jn/118.6.712

[B11] Lei X Stahl C Biotechnological development of effective phytases for mineral nutrition and environmental protection Appl Microb Biot 2001 57 4 474 481 10.1007/s002530100795 11762591

[B12] Lei X Ku PK Miller ER Ullrey DE Yokoyama MT Supplemental microbial phytase improves bioavailability of dietary zinc to weanling pigs J Nutr 1993 123 6 1117 1123 8389400 10.1093/jn/123.6.1117

[B13] Lei XG Ku PK Miller ER Yokoyama MT Ullrey DE Supplementing corn-soybean meal diets with microbial phytase maximizes phytate phosphorus utilization by weanling pigs J Anim Sci 1993 71 12 3368 3375 8294289 10.2527/1993.71123368x

[B14] Sebastian S Touchburn SP Chavez ER Implications of phytic acid and supplemental microbial phytase in poultry nutrition: a review World Poultry Sci J 1998 54 27 47 10.1079/WPS19980003

[B15] Harland BF Oberleas D Phytic acid complex in feed ingredients Phytase in Animal Nutrition and Waste Management: A BASF Reference 1999 Mount Olive: NJ BASF Corp

[B16] Yi Z Kornegay ET Ravindran V Denbow DM Improving phytate phosphorus availability in corn and soybean meal for broilers using microbial phytase and calculation of phosphorus equivalency values for phytase Poultry Sci 1996 75 2 240 249 10.3382/ps.0750240 8833377

[B17] da Silva LG Trugo LC da Costa TS Couri S Low phytate lupin flour based biomass obtained by fermentation with a mutant of *Aspergillus niger* Process Biochem 2005 40 2 951 954 10.1016/j.procbio.2004.02.016

[B18] Vohra A Satyanarayana T Phytase production by the yeast, *Pichia anomala* Biotech Lett 2001 23 7 551 554 10.1023/A:1010314114053

[B19] Vohra A Rastogi SK Satyanarayana T Amelioration in growth and phosphorus assimilation of poultry birds using cell-bound phytase of *Pichia Anomala* World J Microb Biotech 2006 22 6 553 558 10.1007/s11274-005-9070-8

[B20] Yoo GY Wang XJ Choi SY Han K Kang JC Bai SC Dietary microbial phytase increased the phosphorus digestibility in juvenile Korean rockfish Sebastes schlegeli fed diets containing soybean meal Aquaculture 2005 243 1–4 315 322

[B21] Fu DW Huang HQ Luo HY Wang YR Yang PL Meng K Bai YG Wu NF Yao B A highly pH-stable phytase from *Yersinia kristeensenii*: Cloning, expression, and characterization Enzyme Microb Tech 2008 42 6 499 505 10.1016/j.enzmictec.2008.01.014

[B22] In MJ Jang ES Kim YJ Oh NS Purification and properties of an extracellular acid phytase from *Pseudomonas fragi* Y9451 J Microb Biot 2004 14 1004 1008

[B23] Shah P Bhavsar K Soni S Khire J Strain improvement and up scaling of phytase production by *Aspergillus niger* NCIM 563 under submerged fermentation conditions J Ind Microb Biot 2009 36 3 373 380 10.1007/s10295-008-0506-7 19082644

[B24] Simon O Igbasan F *In vitro* properties of phytases from various microbial origins Int J Food Sci Tech 2002 37 7 813 822 10.1046/j.1365-2621.2002.00621.x 11195907

[B25] Shimizu M Purification and characterization of phytase from *Bacillus subtilis* (natto) N-77 Biosci Biotech Biochem 1992 56 8 1266 1269 10.1271/bbb.56.1266

[B26] Nampoothiri K Tomes G Roopesh K Szakacs G Nagy V Soccol CR Pankey A Thermostable phytase production by *Thermoascus aurantiacus* in submerged fermentation Appl Biochem Biotech 2004 118 1 205 214 10.1385/abab:118:1-3:205 15304750

[B27] Pandey A Szakacs G Soccol CR Rodriguez-Leon JA Soccol VT Production, purification and properties of microbial phytases Bioresour Technol 2001 77 3 203 214 10.1016/S0960-8524(00)00139-5 11272007

[B28] Bogar B Szakacs G Linden JC Pandey A Tengerdy RP Optimization of phytase production by solid substrate fermentation J Ind Microbiol Biot 2003 30 3 183 189 10.1007/s10295-003-0027-3 12715256

[B29] Chadha BS Harmeet G Mandeep M Saini HS Singh N Phytase production by the thermophilic fungus *Rhizomucor pusillus* World J Microb Biot 2004 20 1 105 109

[B30] Singh B Kaur P Satyanarayana T Chauhan AK, Verma A **Fungal phytases in ameliorating nutritional status of foods and combating environmental phosphorus pollution** Microbes: Health and Environment 2006 New Delhi, India: IK International Publishers 289 326

[B31] Lassen SF Breinholt J Østergaard PR Brugger R Bischoff A Wyss M Fuglsang CC Expression, gene cloning, and characterization of five novel phytases from four *Basidiomycete* Fungi: *Peniophora lycii, Agrocybe pediades, Ceriporia sp., and Trametes pubescens* Appl Environ Microb 2001 67 10 4701 4707 10.1128/AEM.67.10.4701-4707.2001 PMC93222 11571175

[B32] Maenz DD Engele-Schaan CM Newkirk RW Classen HL The effect of minerals and mineral chelators on the formation of phytase-resistant and phytase-susceptible forms of phytic acid in solution and in a slurry of canola meal Anim Feed Sci Tech 1999 81 3–4 177 192

[B33] Oh BC Choi WC Park S Kim YO Oh TK Biochemical properties and substrate specificities of alkaline and histidine acid phytases Appl Microb Biotech 2004 63 4 362 372 10.1007/s00253-003-1345-0 14586576

[B34] Wyss M Brugger R Kronenberger A Rémy R Fimbel R Oesterhelt G Lehmann M van Loon APGM Biochemical characterization of fungal phytases (myo-inositol hexakisphosphate phosphohydrolases): catalytic properties Appl Environ Microb 1999 65 2 367 373 10.1128/aem.65.2.367-373.1999 PMC91034 9925555

[B35] Cao L Wang WM Yang CT Yang Y Diana J Yakupitiyage A Luo Z Li DP Application of microbial phytase in fish feed Enzyme Microb Tech 2007 40 4 497 507 10.1016/j.enzmictec.2007.01.007

[B36] Kerovuo J Lappalainen I Reinikainen T The metal dependence of *Bacillus subtilis* phytase Biochem Biophy Res Comm 2000 268 2 365 369 10.1006/bbrc.2000.2131 10679209

[B37] Kim DH Oh BC Choi WC Lee JK Oh TK Enzymatic evaluation of *Bacillus amyloliquefaciens* phytase as a feed additive Biotech Lett 1999 21 11 925 927 10.1023/A:1005602717835

[B38] Boyce A Walsh G Purification and characterisation of an acid phosphatase with phytase activity from *Mucor hiemalis Wehmer* J Biotech 2007 132 1 82 87 10.1016/j.jbiotec.2007.08.028 17889394

[B39] Fujita J Yamane YI Fukuda H Kizaki Y Wakabayashi S Shigeta S Suzuki O Ono K Production and properties of phytase and acid phosphatase from a sake koji mold, *Aspergillus oryzae* J Biosci Bioeng 2003 95 4 348 353 16233418 10.1016/s1389-1723(03)80066-x

[B40] Greiner R Konietzny U Phytase for food application Food Tech Biotech 2006 44 2 125 140

[B41] Quan CS Tian WJ Fan SD Kikuchi JI Purification and properties of a low- molecular-weight phytase from *Cladosporium* sp. FP-1 J Biosci Bioeng 2004 97 4 260 266 16233625 10.1016/S1389-1723(04)70201-7

[B42] In MJ Seo SW Kim DC Oh NS Purification and biochemical properties of an extracellular acid phytase produced by the *Saccharomyces cerevisiae* CY strain Process Biochem 2009 44 1 122 126 10.1016/j.procbio.2008.10.006

[B43] Yu P Chen YR Screening and identification of novel phytase-producing *Bacillus* strain from soil J Chin Inst Food Sci Tech 2010 10 116 121

[B44] Laemmli UK Cleavage of structural proteins during the assembly of the head of bacteriophage T4 Nature 1970 227 680 685 10.1038/227680a0 5432063

[B45] Engelen AJ Vander HFC Randsdorp PHG Smit EL Simple and rapid determination of phytase activity J AOAC Int 1994 77 760 764 8012231

[B46] Bradford MM A rapid and sensitive method for the quantization of microgram quantities of protein utilizing the principle of protein-dye binding Anal Biochem 1976 72 248 254 10.1016/0003-2697(76)90527-3 942051

[B47] Fernandez J DeMott M Atherton D Mische SM Internal protein sequence analysis: enzymatic digestion for less than 10 micrograms of protein bound to polyvinylidene difluoride or nitrocellulose membranes Anal Biochem 1992 201 2 255 264 10.1016/0003-2697(92)90336-6 1632512

